# On the misuse of “weighted” composite scores: the scoring procedure of the FEED questionnaire does not indicate whether it is necessary to differentiate between the frequency of experiencing an emotion and the desire to eat in response to that emotion in the assessment of emotional eating

**DOI:** 10.1007/s40519-021-01298-y

**Published:** 2021-09-12

**Authors:** Adrian Meule

**Affiliations:** 1 Department of Psychiatry and Psychotherapy, University Hospital, LMU Munich, Munich, Germany; 2 Schoen Clinic Roseneck, Am Roseneck 6, 83209 Prien am Chiemsee, Germany

Recently, Cassioli et al. [1] developed the Florence Emotional Eating Drive (FEED) questionnaire for the assessment of emotional eating. In contrast to existing emotional eating questionnaires, the FEED not only assesses how strong the desire to eat is in response to different emotions, but also how often this emotion is experienced in the first place. This procedure may provide valuable information as emotional eating may not be a big issue for someone who has a strong desire to eat in response to certain emotions but rarely experiences these emotions. Thus, considering this frequency may increase validity of emotional eating questionnaires, which do not seem to predict actual food intake reliably [2].

Scores of the frequency and the desire to eat subscale are recorded on 5-point scales ranging from 0 to 4. However, the authors then created a scoring procedure that recodes scores from both subscales to a 10-point scale ranging from 0 to 9. For example, a score of 4 on the frequency scale and a score of 4 on the desire to eat scale would be recoded to 9. Yet, the weighting algorithm remains elusive as it is not a simple summation, averaging, or multiplication of scores. Yet, even if the weighting algorithm would be different, such a scoring procedure does not provide any information whether it is actually necessary to assess the frequency of experiencing an emotion and the desire to eat in response to that emotion separately and whether there should be a weighted combination of scores.

Using composite scores has been criticized decades ago [3], but they are still tenaciously used by researchers. Examples include measures of quality of life [4], rejection sensitivity [5], or intrusion load [6], for which two variables are multiplied to create weighted composite scores. However, such scores create several problems in interpretation and do actually not answer the question that researchers are interested in [7, 8]. As an example, let us consider the correlation between the FEED total score and body mass index (BMI). The coefficient is *r* = 0.14, indicating a small, positive relationship. However, it is unknown whether this relationship is primarily due to the frequency subscale, the desire to eat subscale, or their combination. To test this, the easiest way would be to use moderated regression analysis [9, 10], in which BMI is predicted by three independent variables: frequency scores, desire to eat scores, and their product term (frequency × desire to eat). If the interaction term is significant, this would indicate that the size (or direction) of the relationship between desire to eat scores and BMI depends on frequency scores (or vice versa, that the relationship between frequency scores and BMI depends on desire to eat scores). For example, the nature of the interaction effect may be that a higher desire to eat in response to certain emotions relates to higher BMI but only in those with high frequency scores, showing that the combination of both subscales does indeed provide meaningful information. If the interaction effect is not significant, it is still possible that the frequency and desire to eat subscale have additive effects (i.e., both may independently relate to BMI). However, it may also be that the association between emotional eating and BMI is driven only by one subscale.

To conclude, the way that the FEED questionnaire is constructed may indeed lead to new insights for the assessment of emotional eating. However, the scoring procedure that is currently used does not provide these insights. Instead of creating composite scores, scores of both FEED subscales should be used both separately and interactively in moderated regression analyses or other types of statistical procedures, in which such effects can be tested (e.g., structural equation modeling). Only such analyses can reveal whether it is actually necessary to consider the frequency of how often an emotion is experienced and the desire to eat in response to that emotion separately and in combination. However, such analyses may also reveal that one of these subscales suffices in the assessment of emotional eating.

## Declarations

### Ehtical approval

This article does not report any studies with human participants or animals performed by any of the authors.
